# Trends in liver cancer burden in China and G20: a comparative analysis using GBD 2021

**DOI:** 10.3389/fonc.2025.1642502

**Published:** 2025-10-06

**Authors:** Xiaodi Ma, Lining Jiang, Bing Han, Wenlong Ding, Shaochun Liu, Bing Li, Mingjun Zhang, Senbang Yao

**Affiliations:** ^1^ Heilongjiang University of Traditional Chinese Medicine, Harbin, Heilongjiang, China; ^2^ Harbin Medical University, Harbin, Heilongjiang, China; ^3^ The Affiliated Qingyuan Hospital (Qingyuan People’s Hospital), Guangzhou Medical University, Qingyuan, Guangdong, China; ^4^ Yichun Vocational College, Yichun, Heilongjiang, China; ^5^ Harbin Medical University Cancer Hospital, Harbin, Heilongjiang, China; ^6^ Department of Oncology, The Second Hospital of Anhui Medical University, Hefei, Anhui, China

**Keywords:** liver cancer, China, G20, incidence, mortality, disability-adjusted life years

## Abstract

**Objective:**

To compare liver cancer trends and risk factors in China with those in G20 countries (1990–2021) to inform prevention and resource allocation.

**Methods:**

GBD 2021 data for liver cancer in China and G20 countries (1990–2021) were analyzed. Age-standardized incidence rates (ASIR), age-standardized mortality rates (ASMR), and age-standardized disability-adjusted life-years rates (ASDR) were computed using standard population weights. Joinpoint regression estimates the annual percent change (APC; the year-on-year rate of change within each log-linear segment) and the average annual percent change (AAPC; the summary average trend across the entire period). Decomposition analysis partitioned changes into aging, population growth, and epidemiological factors. Trends in ASMR and ASDR attributable to risk factors from 1990 to 2021 in China and G20 countries were assessed.

**Results:**

In 2021, the ASIR, ASMR, and ASDR of liver cancer in China were 29.05 (95% UI, 22.42–36.20), 21.51 (95% UI, 16.66–26.61), and 501.26 (95% UI, 387.29–627.98) per 100,000 population, respectively, reflecting a decline compared with 1990. In both 1990 and 2021, G20 countries had substantially lower liver cancer–related deaths than China. Joinpoint regression showed a significant overall decline in APC from 1990 to 2021 in both regions, with a more pronounced decline in China during 2000–2005 than in the G20. Decomposition analysis identified population aging as the primary contributor to ASIR, ASMR, and ASDR in both China and G20 countries. However, epidemiological improvements had a greater impact on reducing ASMR and ASDR in China, whereas population growth was the dominant factor in G20 countries. ASMR and ASDR attributable to high BMI and drug use increased in both settings, and rates attributable to high fasting plasma glucose (FPG) rose steadily, particularly in G20 nations.

**Conclusion:**

Although age-standardized liver cancer rates declined in China, the absolute burden rose in both China and G20 countries. Increases in incidence and attributable burden from high BMI, drug use, and high FPG highlight the need for enhanced screening, risk factor control, and targeted prevention.

## Introduction

Liver cancer is a malignant tumor originating from hepatic epithelial cells that pose a serious threat to global human health and has become a primary focus of public health prevention and control efforts worldwide (1). According to GLOBOCAN estimates, 800,000 new cases of liver cancer and 750,000 deaths occurred globally in 2022, accounting for 4.3% of all cancer cases and 7.8% of all cancer deaths, respectively (2). Although the age-standardized incidence rate (ASIR) has displayed a decreasing trend, population aging has driven an increase in the absolute number of cases; it is projected that, by 2040, new cases and deaths will rise by 55.0% and 56.4%, respectively, compared with 2020 (3). East Asia, Southeast Asia, and Eastern Africa represent high-incidence regions. In China, although the age-standardized liver cancer mortality rate has declined overall, it remains higher than that of Japan, the Republic of Korea, and the global average (4). Data released by the Chinese National Cancer Center indicate that, in 2022, 367,700 new cases of primary liver cancer were diagnosed, ranking fourth among all cancers, and the incidence ranked fifth. Notably, 316,500 deaths were attributed to primary liver cancer in 2022, making it the second leading cause of cancer death, following lung cancer (5).

In China, surgical interventions such as hepatectomy and liver transplantation represent potentially curative treatments; however, only 20% to 30% of patients meet surgical criteria, and the 5-year postoperative recurrence rate reaches 40% to 70% (6). Therefore, analyzing trends in liver cancer within China and comparing these trends with those of other countries is critical for informing health policy and medical measures. The 2021 Global Burden of Disease (GBD 2021) study is a publicly accessible, comprehensive database that records the burden of more than 300 diseases and injuries across 204 countries and regions (7). While the GBD 2021 provides cross-national data support for liver cancer analysis, the existing literature has primarily focused on global trends (8), specific regions such as Asia-Oceania (9), or specific age groups, including childhood and adolescence (8). However, there is a paucity of research comparing liver cancer trends between China and other countries, and relatively few studies have explored heterogeneity across different countries and regions. This gap hinders China’s ability to identify deficiencies in its health policies and learn from, and implement, the effective strategies employed by other nations.

The Group of Twenty (G20) is an international forum comprising 19 countries and the European Union. Its members are Argentina, Australia, Brazil, Canada, China, France, Germany, India, Indonesia, Italy, Japan, Mexico, the Republic of Korea, the Russian Federation, Saudi Arabia, South Africa, Turkey, the United Kingdom, and the United States, plus the European Union (9). G20 members account for 85% of global gross domestic product and represent diverse health systems. Notably, the Republic of Korea serves as a model for hepatitis B virus (HBV) control, having achieved a 99.4% completion rate of a three-dose HBV vaccination schedule among newborns in 2013. In contrast, China, the Russian Federation, and India remain countries with high hepatitis C virus (HCV) prevalence (10, 11). Therefore, a comparative analysis of disease burden and long-term trends between China and G20 countries holds practical significance. To date, no study has systematically compared liver cancer burden and long-term trends between China and G20 countries.

Accordingly, this Study utilized GBD 2021 data to comprehensively analyze liver cancer burden and attributable risk factors between China and G20 countries from 1990 to 2021. Joinpoint regression analysis was employed to examine temporal trends, and changes over the past three decades were assessed by age and sex. The objectives were to evaluate the liver cancer burden and differences between China and G20 countries, informing targeted prevention strategies and promoting equitable allocation of public health resources.

## Methods

### Data source

This study constituted a secondary analysis of de-identified, aggregated data published in GBD 2021. The Global Burden of Disease study provides comprehensive disease burden data from 1990 to 2021, covering 204 countries and regions and 811 subregions worldwide. GBD 2021 records mortality rates and years of life lost (YLL) for 288 causes of death and includes information stratified by age, sex, and location (12). For the present study, data on liver cancer were obtained from the Global Health Data Exchange (GHDx) platform for the period 1990–2021 for China and G20 countries, encompassing all age groups from 0–4 years to 95+ years, as well as age-standardized metrics. The datasets included incidence, mortality, and disability-adjusted life years (DALYs). Liver cancer cases were identified by the International Classification of Diseases, Tenth Revision (ICD-10) code C22.

### Analysis of trends in China and G20 countries

Liver cancer incidence, mortality, and DALY data, along with corresponding ASIR, the age-standardized mortality rate (ASMR), and age-standardized DALYs rate (ASDR) for China and G20 countries, were extracted from GBD 2021. Age standardization was performed by weighting the incidence or mortality rate for each age group according to a standard population structure, thereby providing a uniform rate that removes the influence of age distribution differences on comparative results (13). The age-standardized rate (ASR) was calculated using the formula:


$$ASR=\frac{\sum_{i=1}^{n}\left(r_{i}\times w_{i}\right)}{\sum_{i=1}^{n}w_{i}}$$

In this context, $r_{i}$ represents the incidence or mortality rate for the i -th age group and $w_{i}$ denotes the weight of the corresponding age group in the standard population. To assess the reliability of the estimates, we calculated the 95% confidence intervals (95% CI) for each indicator.

Trends in ASIR, ASMR, and ASDR from 1990 to 2021 were analyzed using Joinpoint regression models to calculate the annual percent change (APC) and the average annual percent change (AAPC), each with 95% confidence intervals (CIs). The AAPC was calculated as:


AAPC=(exp(β)−1)×100%

Within each time segment between joinpoints, we fit a log-linear model ln(ASR_t_) = α_j_ + β_j_·t, where β_j_ is the slope estimated from regressing ln(ASR) on calendar year t. β_j_ represents the instantaneous log-relative change in ASR per year; equivalently, exp(β_j_) is the multiplicative change in ASR for a one-year increase in t, yielding APC_j_ = (exp(β_j_) − 1) × 100%. For the overall period (1990–2021), β= ∑w_j_β_j_/∑w_j_ is the length-weighted average of segment slopes (with w_j_ equal to years in segment j). When no joinpoints are present, βreduces to β and AAPC equals the single-segment APC. An AAPC > 0 indicates an increasing trend, whereas an AAPC< 0 indicates a decreasing trend (14). If the 95% confidence interval for the AAPC includes 0, the change is considered not statistically significant.

### Decomposition analysis

Changes in the burden of liver cancer from 1990 to 2021 were further investigated using Das Gupta decomposition analysis (15). This method disaggregates overall changes into contributions from demographic aging, population growth, and epidemiological change, thereby elucidating how each factor influences variations in the liver cancer burden.

### Risk factor analysis

Four major risk factors—drug use, high body-mass index (BMI), high fasting plasma glucose (FPG), and smoking—were identified and analyzed to evaluate temporal trends in ASMR and ASDR attributable to these factors in China and G20 countries from 1990 to 2021.

All data processing, analysis, and visualization were conducted using R software (version 4.4.1); statistical significance was defined as <0.05.

## Results

### Overall trends of liver cancer incidence, mortality, and DALYs in China and G20 countries

From 1990 to 2021, the number of incident liver cancer cases in China increased from 96,434.35 (95% UI: 79,381.43–115,868.63) to 196,636.59 (95% UI: 153,913.32–249,394.07). Over the same period, cumulative deaths rose from 94,937.12 (95% UI: 78,251.57–113,898.37) to 172,068.40 (95% UI: 135,402.24–217,746.15), and DALYs increased from 3,294,864.31 (95% UI: 2,700,936.81–3,979,743.64) to 4,890,023.03 (95% UI: 3,804,389.94–6,269,599.56). Despite these increases in case numbers, ASIR, ASMR, and ASDR all demonstrated declining trends. Between 1990 and 2021, the AAPC for ASIR was –0.32 (95% CI: –0.39 to –0.24), for ASMR was –0.79 (95% CI: –1.21 to –0.37), and for ASDR was –0.97 (95% CI: –1.45 to –0.49).

In contrast, G20 countries experienced an increase in liver cancer cases from 185,549.17 (95% UI: 165,956.47–206,120.79) in 1990 to 407,346.54 (95% UI: 353,084.78–468,138.09) in 2021. During the same interval, cumulative deaths increased from 178,992.47 (95% UI: 159,775.82–198,995.02) to 361,134.17 (95% UI: 313,342.08–412,748.32), and DALYs rose from 5,609,295.29 (95% UI: 4,955,812.63–6,320,742.29) to 9,250,469.06 (95% UI: 8,010,177.85–10,744,183.45). From 1990 to 2021, the AAPC for ASIR in G20 countries was 0.22 (95% CI: 0.18 to 0.27), for ASMR was –0.10 (95% CI: –0.32 to 0.12), and for ASDR was –0.46 (95% CI: –0.77 to –0.16). Notably, in both 1990 and 2021, G20 countries’ age-standardized death rates were significantly lower than those in China (**Table 1**).

**Table 1:** All-ages cases, age-standardized incidence, mortality, DALY rates, and corresponding AAPC of liver cancer in China and G20 countries in 1990 and 2021.

	1990			2021		
Location	Measure	All-ages cases (95 % UI)	Age-standardized rates per 100,000 people (95 % UI)	All-ages cases (95 % UI)	Age-standardized rates per 100,000 people (95 % UI)	1990-2021 AAPC (95 % CI)
	Incidence	96434.35 (79381.43 to 115868.63 )	10.58 (8.72 to 12.67 )	196636.59 (153913.32 to 249394.07 )	9.51 (7.44 to 12.06 )	-0.32 (-0.39 to -0.24)
China	Mortality	94937.12 (78251.57 to 113898.37 )	10.75 (8.88 to 12.86 )	172068.40 (135402.24 to 217746.15 )	8.35 (6.57 to 10.56 )	-0.79 (-1.21 to -0.37)
	DALYs	3294864.31 (2700936.81 to 3979743.64 )	334.46 (274.46 to 403.03 )	4890023.03 (3804389.94 to 6269599.56 )	239.62 (186.28 to 307.48 )	-0.97 (-1.45 to -0.49)
	Incidence	185549.17 (165956.47 to 206120.79 )	5.89 (5.26 to 6.52 )	407346.54 (353084.78 to 468138.09 )	6.32 (5.47 to 7.28 )	0.22 (0.18 to 0.27)
G20	Mortality	178992.47 (159775.82 to 198995.02 )	5.75 (5.13 to 6.38 )	361134.17 (313342.08 to 412748.32 )	5.59 (4.83 to 6.39 )	-0.1 (-0.32 to 0.12)
	DALYs	5609295.29 (4955812.63 to 6320742.29 )	171.25 (151.50 to 192.52 )	9250469.06 (8010177.85 to 10744183.45 )	146.16 (126.22 to 170.22 )	-0.46 (-0.77 to -0.16)

DALYs, disability−adjusted life years; AAPC, average annual percentage change; CI, confidence interval; UI, uncertainty interval.

### Joinpoint regression analysis of liver cancer burden in China and G20 countries

**Figure 1:**
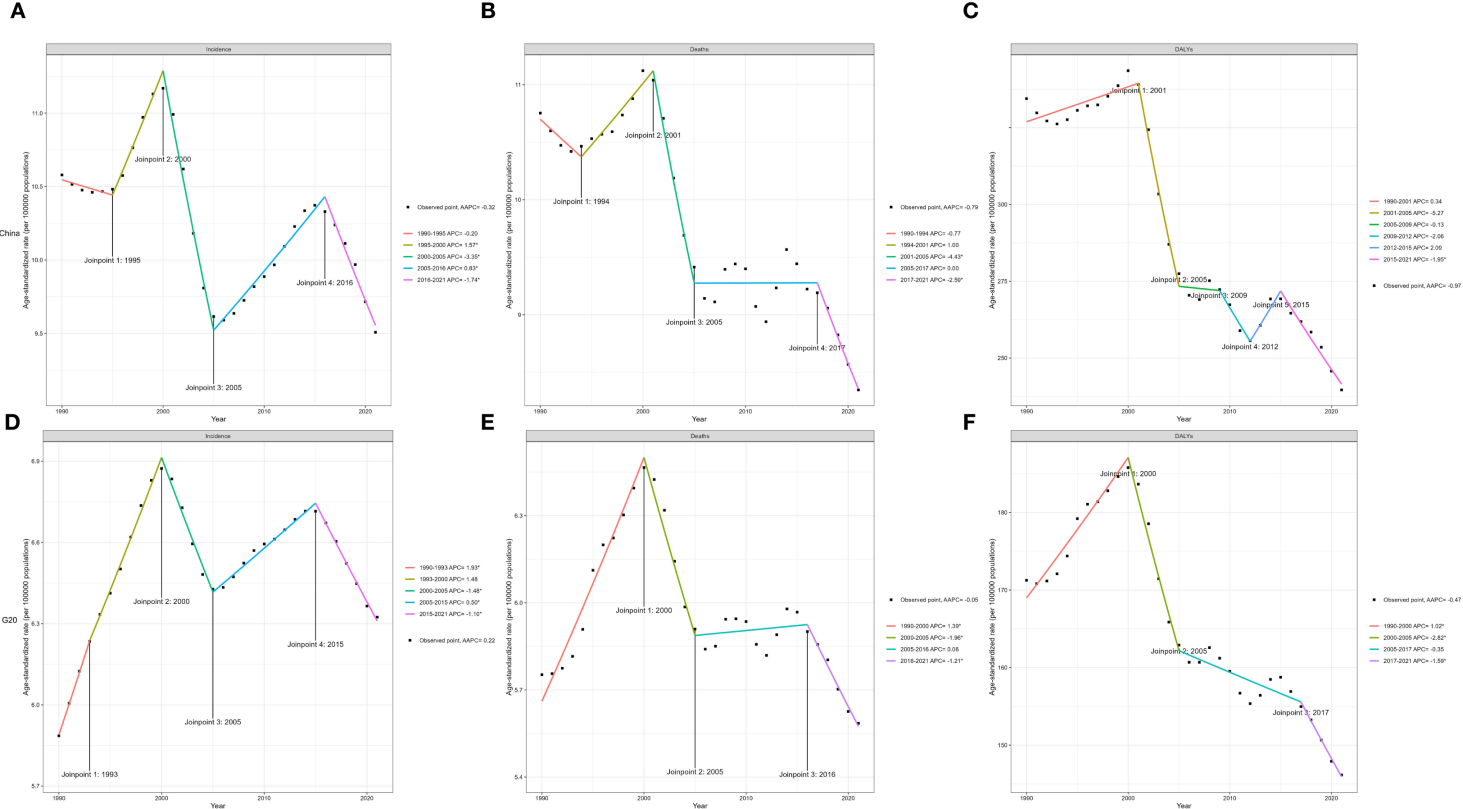
Joinpoint regression analysis of ASIR **(A)**, ASMR **(B)**, and ASDR **(C)** of liver cancer in China from 1990 to 2021. Joinpoint regression analysis of ASIR **(D)**, ASMR **(E)**, and ASDR **(F)** of liver cancer in G20 countries from 1990 to 2021 (* indicates a P value less than 0.05). ASIR, age-standardized incidence rate; ASMR, age-standardized mortality rate; ASDR, age-standardized disability-adjusted life-year rate.

Joinpoint regression analysis of liver cancer ASIR, ASMR, and ASDR in China and G20 countries from 1990 to 2021 is illustrated in **Figure 1**. In China, between 1995 and 2000, ASIR increased (APC = 1.57), followed by a sharp decline from 2000 to 2005 (APC = –3.35). From 2005 to 2016, ASIR rose again (APC = 0.83), and from 2016 to 2021, it resumed a downward trend (APC = –1.74). Regarding ASMR, a decrease was observed between 2001 and 2005 (APC = –4.43). ASDR demonstrated a reduction only during 2015–2021 (APC = –1.95).

In G20 countries, ASIR increased from 1990 to 1993 (APC = 1.93), declined from 2000 to 2005 (APC = –1.48), rose again from 2005 to 2015 (APC = 0.50), and resumed decline from 2015 to 2021 (APC = –1.10). ASMR decreased only during 2000–2005 (APC = –1.96), with no significant changes during other intervals. ASDR increased from 1990 to 2000 (APC = 1.02).

### Trends in liver cancer burden by sex in China and G20 countries

**Figure 2:**
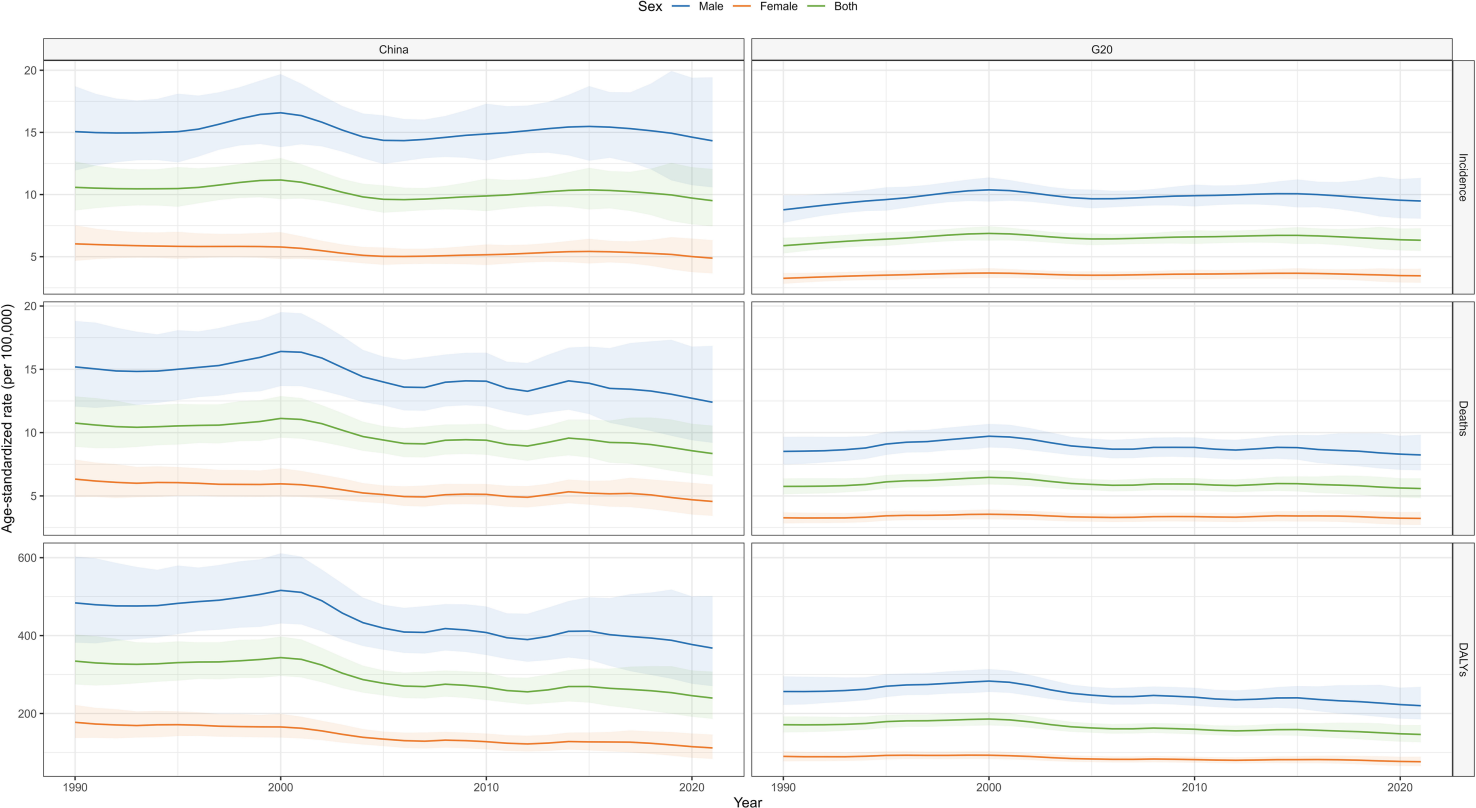
Comparison of the trends in ASIR, ASMR, and ASDR of liver cancer in China and G20.

Between 1990 and 2020, trends in liver cancer burden differed by sex in China and G20 countries. In China, male, female, and overall ASIR initially increased and then declined; the male rate was higher than the female rate and exhibited greater fluctuation. In G20 countries, ASIR remained relatively stable; male rates exceeded female rates but displayed smaller fluctuations. For ASMR, China’s male, female, and overall rates initially rose and then fell; male rates were significantly higher than female rates, peaking around 2000 and then declined. G20 country ASMR trends were flat, with male rates higher than female rates and minimal fluctuation. For ASDR, China’s male, female, and overall rates initially increased and then decreased; male values were higher and more variable. G20 countries showed stable ASDR trends, with male rates exceeding female rates and small fluctuations. Overall, China’s liver cancer–related indicators exhibited greater variability than those of G20 countries, and, in most years, China bore a relatively heavier disease burden. Men consistently exhibited higher rates than women across all indicators (**Figure 2**).

### Comparison of liver cancer burden among different age groups in China and G20 countries

**Figure 3:**
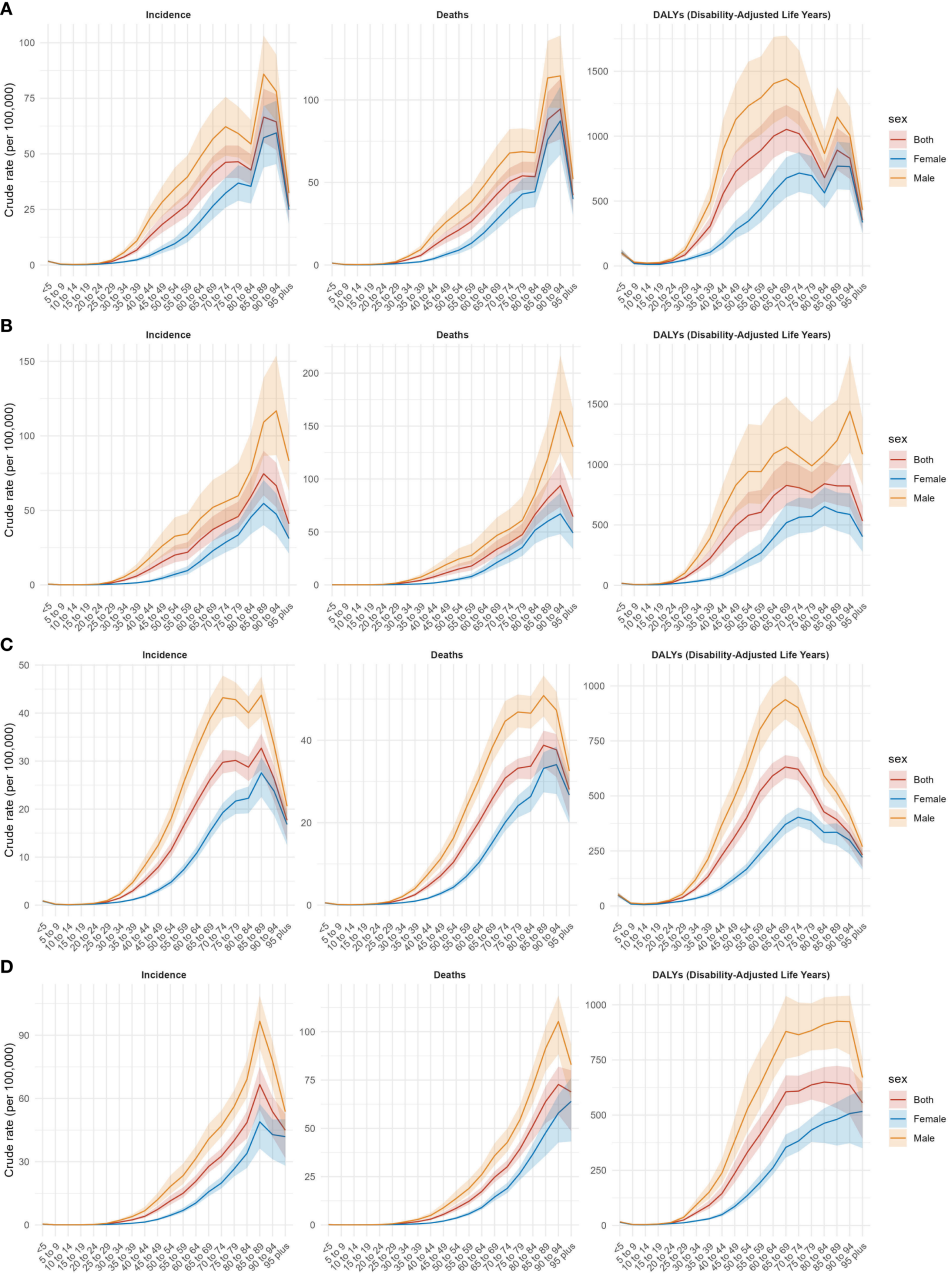
Incidence, mortality, and DALYs for liver cancer by age group in China in 1990 **(A)** and 2021 **(B)**, and in G20 in 1990 **(C)** and 2021 **(D)**.

**Figure 4:**
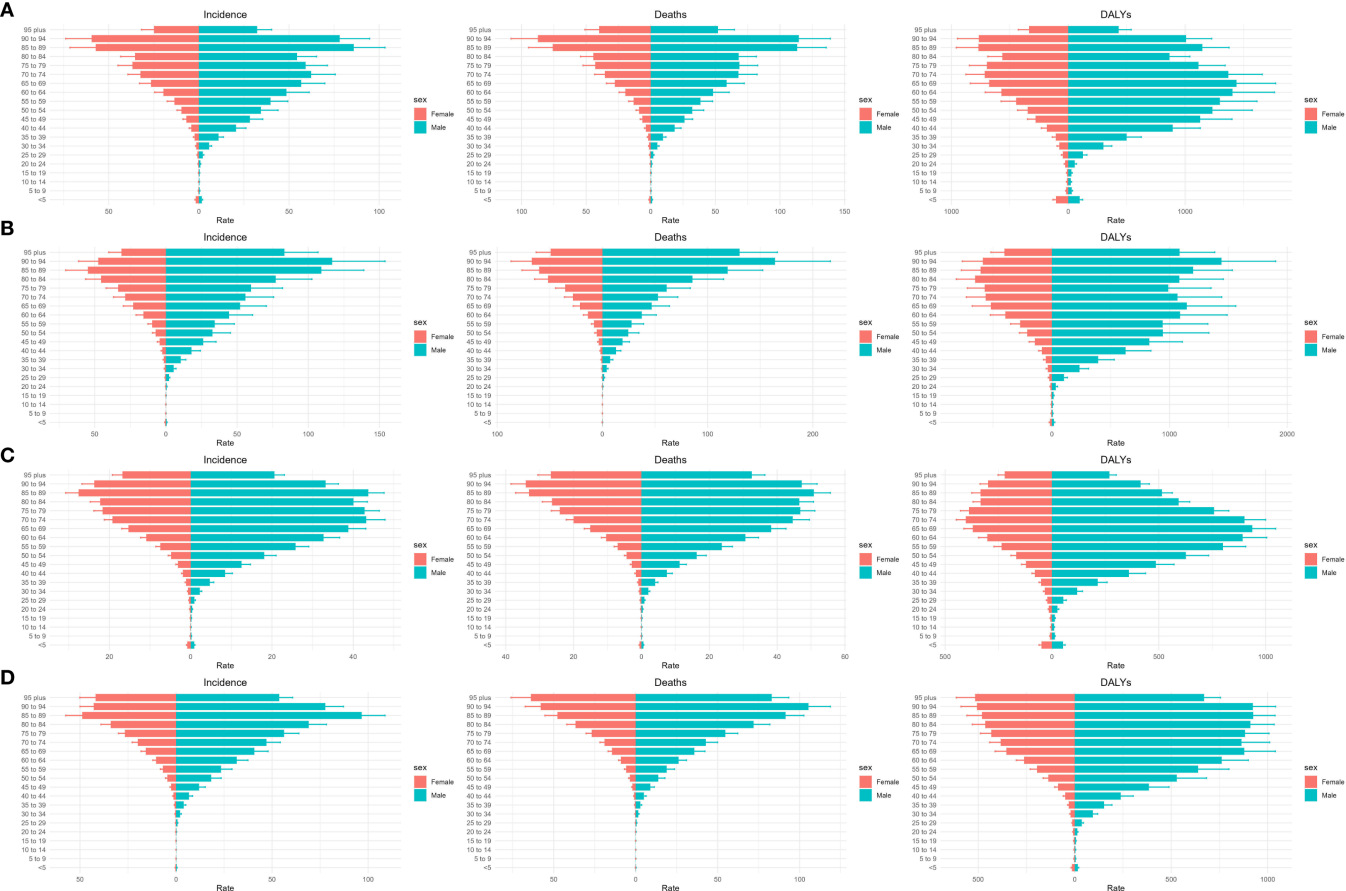
Number of cases, deaths, and DALYs for different age groups of liver cancer in China and G20 in 1990 and 2021. Number of cases, deaths, and DALYs for different age groups in China in 1990 **(A)** and 2021 **(B)**. Number of cases, deaths, and DALYs for different age groups in G20 countries in 1990 **(C)** and 2021 **(D)**. DALYs = disability−adjusted life years.

**Figures 3**, **4** display liver cancer incidence, mortality, DALYs, and deaths by age group in China and G20 countries in 1990 and 2021. In 1990, in China, incidence began to rise in populations older than 35 years; the male incidence peak occurred in the 75–79-year age group, whereas the female peak occurred in those 95 years and older. G20 countries exhibited similar patterns: male incidence peaked at 75–79 years, and female incidence at 95 years and older. By 2021, incidence peaks for both Chinese men and women had shifted to the 95-year-old and older age group; the same shift was observed in G20 countries. Across all age groups, male incidence was generally higher than female incidence.

Regarding mortality, in 1990, both China and G20 countries experienced a marked increase in mortality for those older than 35 years. In China, male and female mortality peaks occurred in higher age brackets; in G20 countries, male and female mortality peaks similarly occurred in higher age brackets, with male mortality exceeding female mortality. By 2021, mortality peaks in both China and G20 countries appeared in the 75–79-year age group for males and the 95-year and older age group for females; male mortality remained higher than female mortality.

For DALYs, in 1990, China’s male DALYs peaked in the 70–74-year age group and then declined; female DALYs also peaked in higher age groups and subsequently declined. In G20 countries, male DALYs peaked in the 70–74-year age group and then declined; female peaks occurred in the 75–79-year age group and declined thereafter. By 2021, male DALYs peaks in both China and G20 countries were observed in the 75–79-year age group, while female peaks were in the 95 years and older age group. Overall, male DALYs exceeded female DALYs. In summary, in both 1990 and 2021, male incidence, mortality, and DALYs in China and G20 countries were higher than those of females across all age groups, and liver cancer–related burdens were markedly elevated among populations older than 35 years. Age-specific incidence, mortality, DALYs, deaths, and corresponding AAPCs are presented in **Table 2**.

**Table 2:** Cases and rates of incidence, mortality, and DALYs of liver cancer in 1990 and 2021 in China and the G20, with AAPC from 1990 to 2021, stratified by age group.

China							G20				
		1990		2021		1990-2021	1990.00		2021.00		1990-2021
Measure	Age Group	Cases (95 % UI)	Rates per 100,000 people (95 % UI)	Cases (95 % UI)	Rates per 100,000 people (95 % UI)	AAPC (95 % CI)	Cases (95 % UI)	Rates per 100,000 people (95 % UI)	Cases (95 % UI)	Rates per 100,000 people (95 % UI)	AAPC (95 % CI)
	<5	1879.68 (1549.57 to 2334.18 )	1.68 (1.39 to 2.09 )	437.81 (303.91 to 622.61 )	0.56 (0.39 to 0.80 )	-3.36 (-4.08 to -2.63)	3203.78 (2673.79 to 3795.97 )	0.86 (0.72 to 1.02 )	1276.05 (1048.51 to 1551.94 )	0.41 (0.33 to 0.49 )	-2.38 (-2.72 to -2.02)
	5 to 9	389.44 (316.65 to 467.61 )	0.37 (0.30 to 0.45 )	115.35 (87.51 to 155.04 )	0.12 (0.09 to 0.16 )	-3.46 (-4.86 to -2.04)	620.56 (544.30 to 704.50 )	0.17 (0.15 to 0.19 )	259.49 (221.70 to 315.55 )	0.07 (0.06 to 0.09 )	-2.71 (-3.26 to -2.17)
	10 to 14	217.92 (178.23 to 267.74 )	0.21 (0.17 to 0.26 )	88.06 (69.46 to 114.84 )	0.10 (0.08 to 0.13 )	-2.42 (-2.73 to -2.11)	313.29 (270.98 to 365.18 )	0.09 (0.08 to 0.11 )	167.56 (142.67 to 198.37 )	0.05 (0.04 to 0.06 )	-2.08 (-2.22 to -1.94)
	15 to 19	348.58 (289.58 to 427.05 )	0.28 (0.23 to 0.34 )	129.65 (104.56 to 166.97 )	0.17 (0.14 to 0.22 )	-1.44 (-1.56 to -1.33)	533.74 (473.20 to 606.43 )	0.15 (0.13 to 0.17 )	333.16 (298.43 to 377.55 )	0.10 (0.09 to 0.11 )	-1.45 (-1.54 to -1.36)
	20 to 24	790.05 (646.45 to 968.09 )	0.60 (0.49 to 0.73 )	308.23 (223.55 to 409.24 )	0.42 (0.31 to 0.56 )	-1.04 (-1.45 to -0.63)	1035.63 (889.59 to 1210.33 )	0.30 (0.26 to 0.35 )	649.57 (573.69 to 757.33 )	0.19 (0.17 to 0.22 )	-1.51 (-1.63 to -1.38)
	25 to 29	1623.15 (1334.90 to 2000.26 )	1.48 (1.21 to 1.82 )	1174.76 (936.33 to 1458.16 )	1.36 (1.08 to 1.69 )	-0.29 (-0.56 to -0.02)	2060.07 (1764.18 to 2453.27 )	0.66 (0.56 to 0.78 )	1822.81 (1573.46 to 2142.09 )	0.52 (0.45 to 0.61 )	-0.84 (-1.1 to -0.57)
	30 to 34	3243.39 (2673.76 to 3891.37 )	3.68 (3.03 to 4.41 )	3879.67 (3037.11 to 5021.22 )	3.20 (2.51 to 4.14 )	-0.44 (-0.59 to -0.29)	4058.27 (3499.25 to 4695.41 )	1.47 (1.27 to 1.70 )	5142.87 (4314.78 to 6289.69 )	1.33 (1.11 to 1.62 )	-0.2 (-0.62 to 0.22)
	35 to 39	6169.97 (5074.46 to 7525.11 )	6.76 (5.56 to 8.24 )	6367.66 (4848.62 to 8334.46 )	6.01 (4.58 to 7.87 )	-0.29 (-0.57 to -0.01)	7747.70 (6611.57 to 9094.15 )	2.97 (2.53 to 3.48 )	8763.77 (7233.02 to 10811.76 )	2.41 (1.99 to 2.97 )	-0.77 (-1.05 to -0.48)
	40 to 44	8586.41 (6932.76 to 10545.07 )	12.80 (10.33 to 15.72 )	9556.41 (7306.14 to 12536.53 )	10.44 (7.98 to 13.70 )	-0.6 (-0.88 to -0.32)	11350.61 (9635.39 to 13346.50 )	5.27 (4.47 to 6.19 )	13538.26 (11144.37 to 16604.87 )	4.10 (3.38 to 5.03 )	-0.79 (-0.88 to -0.7)
	45 to 49	9423.83 (7528.01 to 11469.60 )	18.26 (14.58 to 22.22 )	17092.34 (12848.10 to 22303.37 )	15.49 (11.65 to 20.22 )	-0.47 (-0.72 to -0.23)	13771.42 (11795.03 to 15823.37 )	7.97 (6.83 to 9.16 )	24150.59 (19848.09 to 29634.25 )	7.35 (6.04 to 9.02 )	-0.21 (-0.39 to -0.03)
Incidence	50 to 54	10843.06 (8739.22 to 13274.38 )	22.73 (18.32 to 27.82 )	24228.03 (18315.16 to 32048.97 )	20.05 (15.15 to 26.52 )	-0.37 (-0.61 to -0.13)	18422.82 (16145.91 to 20808.61 )	11.58 (10.15 to 13.08 )	36683.90 (30740.22 to 44727.47 )	11.43 (9.58 to 13.94 )	0 (-0.17 to 0.17)
	55 to 59	11832.59 (9599.09 to 14283.53 )	27.28 (22.13 to 32.93 )	24109.81 (18407.44 to 31540.22 )	21.93 (16.74 to 28.69 )	-0.66 (-0.73 to -0.6)	23536.19 (21078.41 to 26035.59 )	16.69 (14.95 to 18.46 )	43830.56 (37894.73 to 51944.51 )	15.05 (13.01 to 17.83 )	-0.35 (-0.46 to -0.24)
	60 to 64	12214.75 (10171.45 to 14620.43 )	34.57 (28.78 to 41.37 )	22043.02 (17252.82 to 27890.41 )	30.19 (23.63 to 38.20 )	-0.42 (-0.61 to -0.24)	26526.31 (24248.72 to 29019.87 )	21.56 (19.71 to 23.59 )	48727.01 (43770.26 to 55295.27 )	20.75 (18.64 to 23.54 )	-0.13 (-0.24 to -0.03)
	65 to 69	11292.14 (9510.30 to 13330.09 )	41.39 (34.86 to 48.86 )	28564.55 (22713.32 to 35348.13 )	37.24 (29.61 to 46.08 )	-0.34 (-0.64 to -0.03)	25086.40 (23211.67 to 27119.81 )	26.12 (24.17 to 28.24 )	58729.04 (52648.76 to 65970.46 )	27.73 (24.86 to 31.14 )	0.18 (0.06 to 0.3)
	70 to 74	8696.58 (7375.06 to 10119.80 )	46.22 (39.19 to 53.78 )	22276.13 (17903.00 to 27401.24 )	41.80 (33.59 to 51.41 )	-0.31 (-0.4 to -0.22)	19516.50 (18005.36 to 21200.62 )	29.74 (27.44 to 32.31 )	52903.96 (47809.05 to 58763.73 )	32.65 (29.51 to 36.27 )	0.3 (0.16 to 0.43)
	75 to 79	5288.25 (4453.91 to 6117.88 )	46.47 (39.14 to 53.76 )	15206.68 (12436.44 to 18483.27 )	45.92 (37.55 to 55.81 )	-0.02 (-0.08 to 0.03)	14713.14 (13608.05 to 15696.45 )	30.15 (27.89 to 32.17 )	41631.47 (37227.47 to 45659.48 )	40.07 (35.84 to 43.95 )	0.91 (0.8 to 1.01)
	80 to 84	2263.37 (1910.67 to 2639.72 )	42.73 (36.07 to 49.83 )	11739.74 (9671.38 to 14265.17 )	59.32 (48.87 to 72.08 )	1.14 (0.91 to 1.37)	8105.94 (7291.28 to 8724.55 )	28.73 (25.84 to 30.92 )	33989.86 (28677.75 to 37778.52 )	48.62 (41.02 to 54.04 )	1.66 (1.43 to 1.89)
	85 to 89	1122.94 (930.54 to 1337.94 )	66.57 (55.16 to 79.32 )	7105.11 (5725.76 to 8565.98 )	74.59 (60.11 to 89.92 )	0.38 (0.29 to 0.46)	3911.98 (3369.71 to 4274.47 )	32.69 (28.16 to 35.72 )	24803.75 (20322.44 to 27980.75 )	66.59 (54.56 to 75.12 )	2.29 (2.17 to 2.4)
	90 to 94	197.57 (158.74 to 235.42 )	64.39 (51.74 to 76.73 )	1951.46 (1528.89 to 2402.80 )	66.56 (52.15 to 81.95 )	0.18 (-0.11 to 0.47)	894.82 (733.90 to 987.45 )	26.29 (21.57 to 29.02 )	7939.04 (6180.65 to 8989.99 )	53.73 (41.83 to 60.84 )	2.34 (2.22 to 2.46)
	95 plus	10.65 (8.07 to 13.33 )	26.30 (19.93 to 32.91 )	262.12 (193.83 to 325.45 )	41.01 (30.33 to 50.92 )	1.44 (1 to 1.89)	140.01 (106.19 to 158.25 )	17.69 (13.42 to 19.99 )	2003.83 (1414.72 to 2344.48 )	44.94 (31.73 to 52.58 )	3.03 (2.88 to 3.18)
	<5	1267.56 (1045.26 to 1567.36 )	1.13 (0.93 to 1.40 )	140.34 (98.09 to 201.36 )	0.18 (0.13 to 0.26 )	-5.66 (-6.26 to -5.05)	2083.93 (1719.98 to 2495.26 )	0.56 (0.46 to 0.67 )	540.70 (437.36 to 672.71 )	0.17 (0.14 to 0.21 )	-3.73 (-4.07 to -3.38)
	5 to 9	302.55 (245.34 to 364.36 )	0.29 (0.24 to 0.35 )	75.43 (56.87 to 101.01 )	0.08 (0.06 to 0.11 )	-4.03 (-4.91 to -3.13)	478.11 (418.88 to 541.64 )	0.13 (0.12 to 0.15 )	173.39 (146.91 to 212.46 )	0.05 (0.04 to 0.06 )	-3.18 (-3.77 to -2.59)
	10 to 14	229.01 (187.64 to 281.95 )	0.22 (0.18 to 0.28 )	77.64 (61.27 to 100.15 )	0.09 (0.07 to 0.12 )	-2.89 (-3.64 to -2.14)	341.89 (299.42 to 395.47 )	0.10 (0.09 to 0.12 )	171.97 (149.86 to 202.26 )	0.05 (0.04 to 0.06 )	-2.12 (-2.53 to -1.71)
	15 to 19	339.57 (282.13 to 416.54 )	0.27 (0.22 to 0.33 )	103.36 (82.74 to 132.98 )	0.14 (0.11 to 0.18 )	-2.07 (-2.62 to -1.52)	509.69 (450.52 to 580.91 )	0.14 (0.13 to 0.17 )	276.37 (245.19 to 316.15 )	0.08 (0.07 to 0.09 )	-1.93 (-2.26 to -1.59)
	20 to 24	799.34 (654.35 to 974.25 )	0.61 (0.50 to 0.74 )	254.15 (182.73 to 339.14 )	0.35 (0.25 to 0.46 )	-1.57 (-2.47 to -0.67)	1034.12 (886.09 to 1206.49 )	0.30 (0.26 to 0.35 )	556.15 (491.17 to 647.56 )	0.16 (0.14 to 0.19 )	-1.93 (-2.37 to -1.48)
	25 to 29	1500.37 (1234.89 to 1850.44 )	1.37 (1.12 to 1.68 )	885.33 (705.50 to 1104.07 )	1.02 (0.82 to 1.28 )	-0.8 (-2.02 to 0.44)	1880.69 (1606.93 to 2239.69 )	0.60 (0.51 to 0.72 )	1402.00 (1210.87 to 1644.73 )	0.40 (0.34 to 0.47 )	-1.36 (-2.06 to -0.64)
	30 to 34	2956.12 (2442.21 to 3583.06 )	3.35 (2.77 to 4.06 )	2894.71 (2266.73 to 3736.14 )	2.39 (1.87 to 3.08 )	-1.07 (-1.98 to -0.15)	3652.38 (3145.72 to 4259.16 )	1.32 (1.14 to 1.54 )	3897.95 (3252.63 to 4773.28 )	1.01 (0.84 to 1.23 )	-0.61 (-1.97 to 0.78)
	35 to 39	5335.19 (4393.82 to 6481.86 )	5.84 (4.81 to 7.10 )	4515.66 (3428.39 to 5902.92 )	4.26 (3.24 to 5.57 )	-0.95 (-1.61 to -0.3)	6596.95 (5629.85 to 7736.36 )	2.53 (2.16 to 2.96 )	6305.21 (5188.03 to 7747.54 )	1.73 (1.43 to 2.13 )	-1.28 (-1.87 to -0.7)
	40 to 44	7739.89 (6231.30 to 9496.36 )	11.54 (9.29 to 14.15 )	6890.85 (5270.10 to 9106.56 )	7.53 (5.76 to 9.95 )	-1.28 (-1.76 to -0.8)	10006.38 (8454.40 to 11810.54 )	4.64 (3.92 to 5.48 )	9944.94 (8213.80 to 12171.21 )	3.01 (2.49 to 3.69 )	-1.33 (-1.7 to -0.96)
	45 to 49	8689.53 (6932.81 to 10580.57 )	16.83 (13.43 to 20.50 )	12583.03 (9471.20 to 16472.98 )	11.41 (8.59 to 14.93 )	-1.21 (-1.62 to -0.79)	12353.04 (10587.42 to 14258.19 )	7.15 (6.13 to 8.25 )	18005.72 (14820.26 to 22002.37 )	5.48 (4.51 to 6.70 )	-0.82 (-1.07 to -0.56)
Mortality	50 to 54	10145.18 (8173.20 to 12413.23 )	21.26 (17.13 to 26.02 )	18235.07 (13794.75 to 24327.86 )	15.09 (11.41 to 20.13 )	-1.08 (-1.69 to -0.45)	16614.97 (14468.07 to 18856.50 )	10.44 (9.09 to 11.85 )	27786.66 (23276.49 to 33853.56 )	8.66 (7.25 to 10.55 )	-0.61 (-0.96 to -0.25)
	55 to 59	11469.19 (9288.62 to 13900.25 )	26.45 (21.42 to 32.05 )	19661.80 (14954.03 to 25651.64 )	17.88 (13.60 to 23.33 )	-1.2 (-1.69 to -0.7)	21693.19 (19310.74 to 24080.17 )	15.38 (13.69 to 17.07 )	35720.43 (30948.33 to 42240.13 )	12.26 (10.63 to 14.50 )	-0.71 (-0.93 to -0.49)
	60 to 64	12159.79 (10124.07 to 14513.15 )	34.41 (28.65 to 41.07 )	18683.09 (14633.15 to 23630.97 )	25.59 (20.04 to 32.37 )	-0.9 (-1.33 to -0.46)	24968.36 (22713.71 to 27436.10 )	20.29 (18.46 to 22.30 )	40788.87 (36475.59 to 46310.10 )	17.37 (15.53 to 19.72 )	-0.47 (-0.61 to -0.32)
	65 to 69	11723.10 (9875.93 to 13828.87 )	42.97 (36.20 to 50.69 )	25914.67 (20666.76 to 32249.52 )	33.79 (26.94 to 42.04 )	-0.68 (-1.05 to -0.3)	24730.72 (22763.26 to 26858.67 )	25.75 (23.70 to 27.97 )	52282.90 (46766.37 to 58698.53 )	24.68 (22.08 to 27.71 )	-0.09 (-0.36 to 0.18)
	70 to 74	9505.65 (8080.94 to 11076.44 )	50.51 (42.94 to 58.86 )	21305.71 (17150.86 to 26169.64 )	39.98 (32.18 to 49.10 )	-0.72 (-1.22 to -0.22)	20189.57 (18503.86 to 21944.34 )	30.77 (28.20 to 33.44 )	48812.57 (44181.05 to 54277.72 )	30.13 (27.27 to 33.50 )	-0.05 (-0.29 to 0.19)
	75 to 79	6147.78 (5177.18 to 7125.67 )	54.02 (45.49 to 62.61 )	15742.95 (12932.05 to 19175.11 )	47.53 (39.05 to 57.90 )	-0.33 (-0.62 to -0.05)	16203.48 (14973.21 to 17333.49 )	33.21 (30.68 to 35.52 )	40973.99 (36639.59 to 44941.09 )	39.44 (35.27 to 43.26 )	0.55 (0.29 to 0.81)
	80 to 84	2835.00 (2404.37 to 3309.14 )	53.52 (45.39 to 62.47 )	13165.42 (10897.45 to 16080.87 )	66.52 (55.06 to 81.25 )	0.75 (-0.24 to 1.75)	9510.53 (8612.28 to 10236.69 )	33.71 (30.53 to 36.28 )	35887.80 (30689.13 to 39641.55 )	51.33 (43.90 to 56.70 )	1.31 (0.84 to 1.78)
	85 to 89	1485.15 (1230.54 to 1767.37 )	88.04 (72.95 to 104.77 )	7776.84 (6284.27 to 9355.87 )	81.64 (65.97 to 98.22 )	-0.13 (-1.49 to 1.25)	4639.93 (4006.82 to 5062.22 )	38.78 (33.49 to 42.31 )	23793.93 (19613.58 to 26724.38 )	63.88 (52.66 to 71.75 )	1.69 (1.32 to 2.05)
	90 to 94	290.02 (233.91 to 346.07 )	94.52 (76.24 to 112.79 )	2750.54 (2158.48 to 3397.32 )	93.81 (73.62 to 115.87 )	0 (-1.01 to 1.01)	1282.45 (1057.12 to 1412.36 )	37.68 (31.06 to 41.50 )	10743.66 (8430.90 to 12103.43 )	72.71 (57.06 to 81.92 )	2.16 (1.88 to 2.43)
	95 plus	17.12 (13.05 to 21.43 )	42.28 (32.23 to 52.92 )	411.81 (306.82 to 510.04 )	64.44 (48.01 to 79.81 )	1.63 (0.18 to 3.11)	222.10 (167.53 to 250.75 )	28.06 (21.16 to 31.68 )	3068.97 (2164.96 to 3567.56 )	68.83 (48.55 to 80.01 )	2.96 (2.71 to 3.21)
	<5	113466.81 (93482.97 to 140184.72 )	101.49 (83.61 to 125.38 )	12669.77 (8817.93 to 18168.37 )	16.31 (11.35 to 23.39 )	-5.63 (-6.24 to -5.02)	186169.23 (153993.48 to 223087.29 )	50.25 (41.56 to 60.21 )	48536.21 (39268.00 to 60419.51 )	15.48 (12.52 to 19.27 )	-3.71 (-4.06 to -3.37)
	5 to 9	25249.53 (20487.71 to 30396.38 )	24.21 (19.65 to 29.15 )	6308.38 (4754.14 to 8449.60 )	6.59 (4.96 to 8.82 )	-4.02 (-4.91 to -3.13)	39894.95 (34959.37 to 45256.93 )	10.98 (9.62 to 12.45 )	14482.57 (12266.85 to 17755.76 )	4.10 (3.48 to 5.03 )	-3.18 (-3.77 to -2.58)
	10 to 14	17795.91 (14582.27 to 21913.20 )	17.40 (14.26 to 21.42 )	6032.31 (4760.71 to 7785.46 )	7.00 (5.52 to 9.03 )	-2.89 (-3.64 to -2.14)	26572.14 (23265.28 to 30739.95 )	7.76 (6.79 to 8.97 )	13358.29 (11642.01 to 15715.31 )	3.76 (3.28 to 4.42 )	-2.12 (-2.53 to -1.71)
	15 to 19	24703.09 (20531.93 to 30323.12 )	19.50 (16.21 to 23.94 )	7520.92 (6019.74 to 9672.82 )	10.07 (8.06 to 12.95 )	-2.07 (-2.61 to -1.52)	37084.06 (32782.07 to 42301.79 )	10.55 (9.32 to 12.03 )	20109.89 (17837.52 to 23015.79 )	5.85 (5.19 to 6.69 )	-1.93 (-2.26 to -1.59)
	20 to 24	54306.86 (44454.76 to 66181.64 )	41.14 (33.68 to 50.14 )	17253.81 (12410.56 to 23012.70 )	23.58 (16.96 to 31.45 )	-1.58 (-2.47 to -0.68)	70253.46 (60171.42 to 81978.21 )	20.34 (17.42 to 23.74 )	37760.15 (33376.02 to 43916.63 )	10.98 (9.71 to 12.77 )	-1.93 (-2.37 to -1.49)
	25 to 29	94751.85 (77982.01 to 116848.09 )	86.22 (70.96 to 106.33 )	55727.60 (44435.20 to 69514.94 )	64.44 (51.38 to 80.38 )	-0.81 (-2.02 to 0.41)	118721.22 (101457.46 to 141504.37 )	37.90 (32.39 to 45.18 )	88298.50 (76234.52 to 103580.18 )	24.99 (21.58 to 29.31 )	-1.37 (-2.06 to -0.66)
	30 to 34	170902.34 (141142.49 to 207004.84 )	193.67 (159.95 to 234.58 )	167745.63 (131352.79 to 216527.62 )	138.46 (108.42 to 178.72 )	-1.06 (-1.98 to -0.14)	211336.67 (182213.45 to 246541.15 )	76.65 (66.09 to 89.42 )	226004.39 (188578.25 to 276639.89 )	58.36 (48.70 to 71.44 )	-0.6 (-1.95 to 0.77)
	35 to 39	283073.22 (233096.14 to 344227.83 )	309.91 (255.20 to 376.87 )	240212.83 (182401.47 to 314102.05 )	226.69 (172.14 to 296.43 )	-0.94 (-1.6 to -0.28)	350071.58 (298570.03 to 410731.15 )	134.05 (114.33 to 157.27 )	335255.73 (276196.16 to 412149.15 )	92.09 (75.87 to 113.21 )	-1.27 (-1.85 to -0.69)
	40 to 44	372631.96 (300120.52 to 457273.08 )	555.39 (447.31 to 681.54 )	331708.85 (253324.23 to 438612.25 )	362.39 (276.76 to 479.18 )	-1.29 (-1.77 to -0.8)	481782.02 (407063.68 to 568924.87 )	223.59 (188.91 to 264.03 )	478860.44 (395493.16 to 586003.68 )	145.16 (119.89 to 177.64 )	-1.33 (-1.7 to -0.96)
	45 to 49	375341.16 (299629.79 to 456906.59 )	727.14 (580.46 to 885.15 )	542902.51 (409195.40 to 711353.96 )	492.11 (370.91 to 644.80 )	-1.21 (-1.64 to -0.78)	533516.56 (457075.53 to 615466.32 )	308.74 (264.50 to 356.16 )	777696.96 (640325.66 to 950663.14 )	236.65 (194.85 to 289.28 )	-0.82 (-1.07 to -0.56)
DALYs	50 to 54	389204.73 (313408.01 to 475920.13 )	815.76 (656.89 to 997.51 )	700993.56 (530079.02 to 934391.56 )	580.01 (438.59 to 773.13 )	-1.07 (-1.66 to -0.48)	637928.46 (555933.06 to 723986.66 )	400.98 (349.44 to 455.07 )	1068936.40 (896535.98 to 1303288.46 )	333.06 (279.34 to 406.08 )	-0.6 (-0.94 to -0.27)
	55 to 59	387156.68 (313430.10 to 469464.13 )	892.70 (722.70 to 1082.49 )	665519.28 (506408.19 to 867735.26 )	605.33 (460.61 to 789.26 )	-1.17 (-1.66 to -0.69)	732233.13 (651814.62 to 813532.87 )	519.21 (462.18 to 576.85 )	1208144.46 (1047038.72 to 1429896.58 )	414.79 (359.48 to 490.92 )	-0.72 (-0.93 to -0.5)
	60 to 64	353895.30 (294579.19 to 422491.13 )	1001.47 (833.62 to 1195.59 )	543011.88 (425973.98 to 687912.41 )	743.80 (583.48 to 942.28 )	-0.91 (-1.33 to -0.5)	726909.92 (661172.65 to 798431.72 )	590.82 (537.39 to 648.95 )	1187910.98 (1064028.88 to 1348027.83 )	505.78 (453.04 to 573.96 )	-0.47 (-0.62 to -0.33)
	65 to 69	287148.54 (242132.67 to 338256.24 )	1052.53 (887.52 to 1239.86 )	634895.01 (506449.13 to 789968.59 )	827.73 (660.27 to 1029.90 )	-0.68 (-1.05 to -0.3)	606602.84 (558669.87 to 658278.64 )	631.65 (581.74 to 685.46 )	1283091.17 (1147975.93 to 1441070.27 )	605.75 (541.96 to 680.33 )	-0.09 (-0.36 to 0.18)
	70 to 74	191789.57 (162873.90 to 223647.38 )	1019.20 (865.54 to 1188.50 )	430680.68 (346764.11 to 530674.11 )	808.09 (650.63 to 995.70 )	-0.71 (-1.19 to -0.24)	407624.65 (374234.34 to 443720.02 )	621.23 (570.34 to 676.24 )	986609.95 (893592.36 to 1099534.15 )	608.95 (551.54 to 678.65 )	-0.04 (-0.28 to 0.2)
	75 to 79	99679.86 (83824.46 to 115590.20 )	875.87 (736.55 to 1015.67 )	254442.92 (208839.99 to 310115.92 )	768.27 (630.58 to 936.37 )	-0.35 (-0.63 to -0.07)	261868.33 (241915.96 to 280496.57 )	536.64 (495.75 to 574.82 )	661791.40 (592615.63 to 726604.90 )	637.05 (570.46 to 699.44 )	0.55 (0.28 to 0.82)
	80 to 84	36021.59 (30541.95 to 41979.72 )	680.02 (576.58 to 792.50 )	166427.51 (137563.82 to 203235.06 )	840.89 (695.05 to 1026.86 )	0.73 (-0.24 to 1.7)	120726.80 (109312.10 to 129979.35 )	427.92 (387.46 to 460.72 )	454289.27 (388000.14 to 501681.09 )	649.81 (554.99 to 717.60 )	1.3 (0.85 to 1.75)
	85 to 89	15055.85 (12470.78 to 17914.82 )	892.54 (739.29 to 1062.02 )	78439.67 (63373.02 to 94408.55 )	823.45 (665.28 to 991.09 )	-0.14 (-1.47 to 1.22)	46921.51 (40571.99 to 51294.58 )	392.15 (339.08 to 428.70 )	240400.27 (197808.39 to 269527.01 )	645.39 (531.05 to 723.59 )	1.69 (1.31 to 2.06)
	90 to 94	2546.20 (2055.78 to 3041.10 )	829.86 (670.02 to 991.16 )	24126.67 (18921.86 to 29736.70 )	822.88 (645.36 to 1014.22 )	-0.01 (-0.99 to 0.99)	11241.28 (9248.63 to 12412.16 )	330.33 (271.77 to 364.73 )	94131.54 (73837.77 to 105863.37 )	637.08 (499.73 to 716.48 )	2.15 (1.88 to 2.42)
	95 plus	143.29 (109.37 to 179.32 )	353.87 (270.10 to 442.84 )	3403.23 (2544.65 to 4221.62 )	532.50 (398.16 to 660.56 )	1.59 (0.14 to 3.06)	1836.49 (1387.60 to 2077.67 )	232.01 (175.30 to 262.47 )	24800.48 (17525.91 to 28830.76 )	556.20 (393.05 to 646.58 )	2.88 (2.63 to 3.12)

### Decomposition analysis

**Figure 5:**
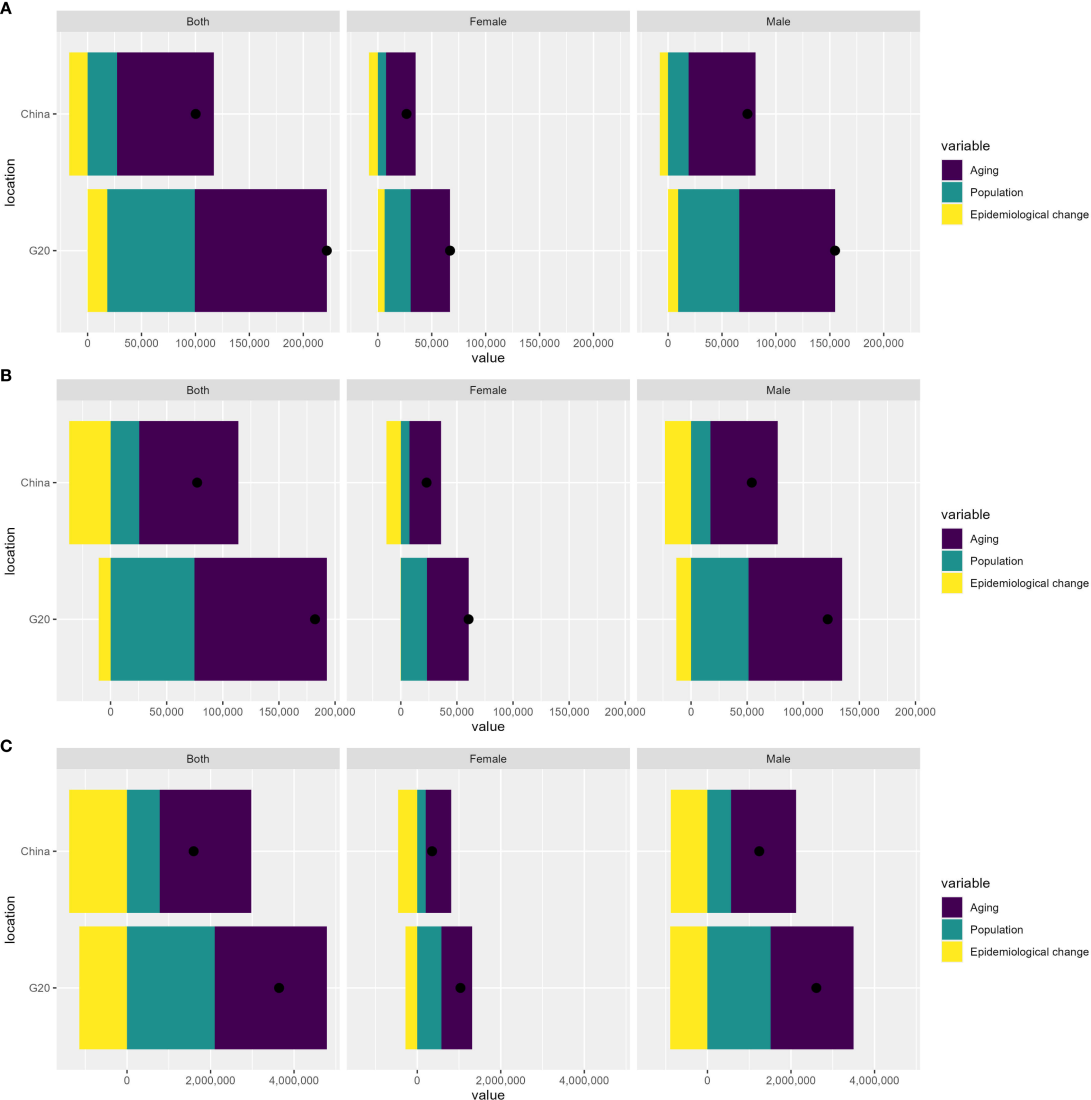
Decomposition analysis of incidence **(A)**, mortality **(B)**, and DALYs **(C)** in China and G20 countries. DALYs, disability-adjusted life years.

**Figure 5** presents a decomposition analysis of incidence, mortality, and DALYs for liver cancer in China and G20 countries. For incidence, mortality, and DALYs, aging made the largest contribution; the liver cancer burden increased with advancing age in both China and G20 countries, representing the primary influencing factor. Notably, for ASIR, the contribution of population growth exceeded that of epidemiological change in both China and G20 countries; however, for ASMR and ASDR, in China, the contribution of population growth was less than that of epidemiological change, whereas in G20 countries, population growth contributed more than epidemiological change. This pattern persisted when stratified by sex (male and female). Changes in incident cases, mortality, and DALYs according to population-level determinants and causes from 1990 to 2021 are detailed in **Supplementary Table S1**.

### Risk factor analysis

**Figure 6:**
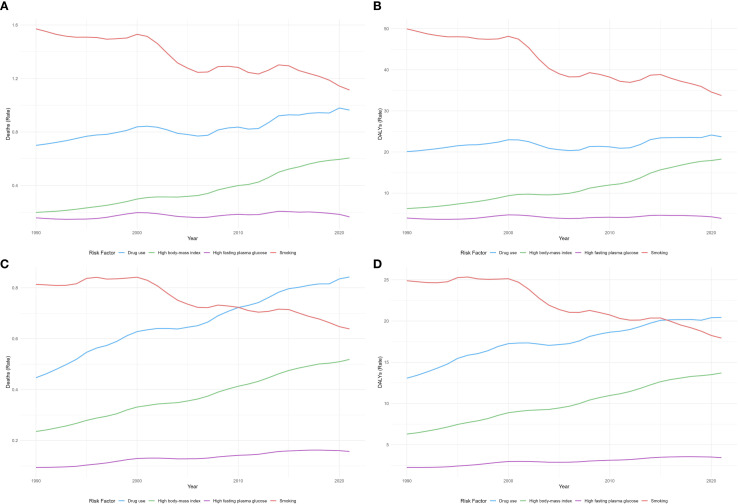
Trends in ASMR **(A)** and ASDR **(B)** of liver cancer attributable to drug use, high BMI, high FPG, and smoking in China from 1990 to 2021. Trends in ASMR **(C)** and ASDR **(D)** of liver cancer attributable to drug use, high BMI, high FPG, and smoking in G20 countries from 1990 to 2021. ASMR, age-standardized mortality rate; ASDR, age-standardized disability-adjusted life-year rate. .

**Figure 6** depicts temporal trends in ASMR and ASDR for liver cancer in China and G20 countries from 1990 to 2021, attributable to drug use, high BMI, high FPG, and smoking. In China, between 1990 and 2020, ASMR attributable to smoking declined, whereas mortality due to high BMI increased. Mortality attributed to drug use rose gradually, and mortality associated with high FPG steadily increased. Regarding ASDR, the rate attributable to smoking declined continuously, while high BMI–related ASDR increased consistently. Drug use–related ASDR rose slowly, and high FPG–related ASDR steadily increased. In G20 countries, ASMR initially attributable to smoking exceeded that due to high BMI; over time, smoking-related mortality began to decline, and high BMI–related mortality rose gradually. Mortality associated with drug use increased steadily, and mortality linked to high FPG steadily increased. For ASDR, smoking-related rates declined, whereas high BMI–related rates increased. Drug use–related ASDR rose slowly, and high FPG–related ASDR steadily increased. Overall, in both China and G20 countries, smoking was associated with declining liver cancer mortality and ASDR; high BMI was positively associated with both; the impact of drug use increased gradually; and high FPG–related ASMR and ASDR increased year by year.

## Discussion

Liver cancer was confirmed to remain a significant public health problem in both China and G20 countries. In this study, GBD 2021 data were utilized for the first time to compare trends in liver cancer incidence, mortality, and DALYs across different age groups and sexes in China and the G20 countries from 1990 to 2021. The analysis aimed to identify attributable risk factors for DALYs and provide guidance for liver cancer prevention and control.

Between 1990 and 2021, absolute numbers of liver cancer cases, deaths, and DALYs increased substantially in both China and G20 countries; however, age-standardized rates (ASIR, ASMR, ASDR) followed divergent trajectories. In China, age-standardized indicators declined across the board, whereas in G20 countries, ASIR increased slightly and ASMR and ASDR declined only modestly. Notably, despite improvements in China’s age-standardized mortality, its absolute age-standardized death rate remained higher than the G20 average. The GBD 2019 data have indicated that age-standardized rates in East Asia (including China) likely declined due to widespread HBV vaccination and the uptake of antiviral therapy (16), consistent with the negative growth trends in ASIR and ASMR observed in this study. By contrast, the increase in ASIR among G20 countries may have been driven by the rising prevalence of metabolic dysfunction-associated fatty liver disease (MAFLD) and alcoholic liver disease in regions such as the United States (17). Although ASMR declined overall in G20 countries, the rate of decline was lower than in China, possibly reflecting China’s recent expansion of early screening programs for liver cancer (e.g., serum alpha-fetoprotein [AFP] combined with ultrasound) (18).

Joinpoint regression showed phase-specific fluctuations in China and the G20. From 2000 to 2005, ASIR, ASMR, and ASDR declined in both settings, with steeper falls in China, as shown in **Figure 1**. Considering the latency between infant hepatitis B vaccination and adult liver cancer, the program is unlikely to be the sole explanation for the decline from 2000 to 2005. A limited contribution is plausible through reduced perinatal and early childhood transmission, as coverage expanded from the early 1990s and was integrated into the national schedule in 2002, with documented declines in HBsAg prevalence among children during the 2000s (19, 20). Additional factors plausibly driving the decline include substantial reductions in dietary aflatoxin exposure in high-risk regions such as Qidong, China, with subsequent reductions in liver cancer mortality (21), early clinical uptake of nucleoside and nucleotide analogues for chronic hepatitis B that lower the risk of hepatocellular carcinoma among treated patients (22), and improvements in blood safety and perinatal prevention including timely birth dose and hepatitis B immunoglobulin (20).

From 2005 to 2015, ASIR rose slightly in China and the G20, with a more pronounced increase in China, while ASMR and ASDR showed little overall change in both settings. This pattern is consistent with, and may be partly related to, the expanding metabolic burden. By 2014, the global age-standardized prevalence of obesity reached 10.8 per cent in men and 14.9 per cent in women, and diabetes reached 9.0 per cent in men and 7.9 per cent in women (23, 24). Excess body weight is associated with higher liver cancer risk, with relative risks of 1.17 for overweight and 1.89 for obesity compared with normal weight (25). A key pathway is metabolic dysfunction-associated steatotic liver disease (MASLD), which is associated with a hazard ratio of 1.88 for hepatocellular carcinoma in a meta-analysis (26) and is an increasingly important etiology across regions (17).

Furthermore, the accelerated decline in China’s ASDR after 2015 may have been related to the Healthy China Action—Cancer Control Implementation Plan (2019–2022), which promoted early screening for liver cancer (27). Conversely, the rise in ASDR in G20 countries from 1990 to 2000 may reflect historical deficiencies in hepatitis screening and antiviral treatment coverage in countries such as India and Indonesia (28). It is also noteworthy that China’s ASMR improvement was concentrated in 2001–2005, whereas G20 countries experienced only a transient decline during the same period, possibly because China implemented multidisciplinary treatment (MDT) models and included targeted therapies in insurance coverage earlier (29).

The sex-specific analysis demonstrated significant differences in liver cancer burden between males and females in both regions from 1990 to 2021. Male ASIR, ASMR, and ASDR were substantially higher than female rates, and fluctuations were more pronounced in China. In China, male liver cancer indicators followed a “rise-then-fall” pattern, whereas trends in G20 countries were relatively stable. Age-stratified data showed that liver cancer burden markedly increased in populations older than 35 years; the peak age for males shifted upward over time but remained older than that for females. Additionally, both absolute burden and age-standardized rates were higher in China than in G20 countries, although China’s declines were more pronounced. Fluctuations in China’s male incidence may have been driven by periodic rebounds in HBV infection rates (30), while high male prevalence of alcohol use and tobacco smoking further widened sex differences (31). By contrast, sex disparities in G20 countries were more stable, perhaps due to higher HCV cure rates offset by increasing metabolic disease risk (31). In China, a persistent high burden among those older than 75 years may reflect the natural history of HBV infection progressing from cirrhosis to carcinoma over decades (32). These findings suggest that, in China, enhanced surveillance among older adults and targeted screening for high-risk male subpopulations are warranted.

Decomposition analysis revealed distinct sociodemographic and epidemiological drivers of liver cancer burden in China and G20 countries. Aging emerged as the primary contributor to ASIR, ASMR, and ASDR in both regions. Importantly, population growth had a stronger impact on ASIR than epidemiological change in both China and G20 countries. However, for ASMR and ASDR, epidemiological improvements (e.g., risk factor control or treatment advances) had a greater influence in China, whereas population growth remained the predominant driver in G20 countries. These differences likely reflect regional variations in prevention strategies. For example, China’s greater epidemiological impact on ASMR/ASDR may be attributable to aggressive HBV vaccination programs and aflatoxin control policies implemented since the 2000s (33), whereas G20 countries’ reliance on population growth reflects slower declines in metabolic risk factors (e.g., obesity, alcohol use) and uneven access to HCV treatment (34). Additionally, China’s accelerated aging (driven by the one-child policy and increased life expectancy) has exacerbated age-related liver cancer susceptibility, whereas G20 countries face complex aging and migration dynamics that influence population growth (35).

Risk factor analysis demonstrated significant declines in smoking-related burden in both China and G20 countries, likely owing to tobacco control policies. In contrast, high BMI emerged as a leading risk factor with increasing impact—consistent with projections indicating that, by 2030, high BMI–related age-standardized mortality in China will rise substantially (36). It is noteworthy that liver cancer attributable to drug use has increased gradually in both regions, more markedly in G20 countries, potentially reflecting opioid abuse in some G20 member states (37). China’s increase may be driven by hepatotoxicity from traditional herbal medicines (38). Furthermore, the impact of high FPG on liver cancer burden has grown over time in G20 countries. In contrast, in China, diabetes has remained relatively unchanged, possibly reflecting China’s public health management (39).

Several limitations warrant consideration. First, reliance on GBD 2021 data may have introduced bias from underreporting or misclassification in source registries, and model‐based adjustments for missing data could contribute additional uncertainty. Second, only national‐level analyses were performed, precluding assessment of subnational variations in disease burden and risk factors. Third, etiologic attribution was restricted to four major risk factors, potentially overlooking other contributors such as environmental toxins or HDV infection. Finally, Joinpoint regression did not account for changes in diagnostic criteria or screening practices over time, which may have influenced observed trends.

## Conclusions

This study offers a detailed comparison of liver cancer burden in China and G20 countries from 1990 to 2021; it reveals that although ASIR, ASMR, and ASDR declined in China, absolute numbers increased in both China and G20 nations. In G20 countries, ASIR increased slightly; ASMR and ASDR decreased modestly, in contrast to China’s more pronounced decline in ASMR. Notably, China’s absolute mortality and ASMR remain higher than G20 averages, underscoring the need for sustained HBV vaccination, antiviral therapy, and early screening. In both China and G20 countries, attention must be paid to the impact of high BMI and drug use on liver cancer burden; G20 nations, in particular, should monitor the gradual rise in liver cancer burden attributable to high FPG. Future research should prioritize subnational analyses to uncover regional disparities and evaluate the impact of evolving screening practices.

## Data Availability

The original contributions presented in the study are included in the article/**Supplementary Material**. Further inquiries can be directed to the corresponding authors.
