# Supplementary material for: Trends in liver cancer burden in China and G20: a comparative analysis using GBD 2021
Source: Front Oncol. 2025 Oct 6;15:1642502. doi: 10.3389/fonc.2025.1642502 (PMC12536301; doi:10.3389/fonc.2025.1642502)
Supplement: Supplementary file 1 [file Supplementaryfile1.docx]

Table S1. Changes in incident cases of incidence, mortality, DALYs according to population-level determinants and causes from 1990 to 2021.

| location | sex | measure | Overll difference | Aging | Population | Epidemiological change |
| --- | --- | --- | --- | --- | --- | --- |
| China | Male | Incidence | 73579.03 | 62156.63 (84.48%) | 19052.75 (25.89%) | -7630.35 (-10.37%) |
| China | Female | Incidence | 26623.21 | 27165.74 (102.04%) | 7756.08 (29.13%) | -8298.61 (-31.17%) |
| China | Both | Incidence | 100202.24 | 89832.07 (89.65%) | 27314.49 (27.26%) | -16944.31 (-16.91%) |
| G20 | Male | Incidence | 154831.85 | 88684.89 (57.28%) | 56646.69 (36.59%) | 9500.28 (6.14%) |
| G20 | Female | Incidence | 66965.52 | 36536.22 (54.56%) | 24176.08 (36.1%) | 6253.21 (9.34%) |
| G20 | Both | Incidence | 221797.37 | 122354.27 (55.16%) | 81145.28 (36.59%) | 18297.82 (8.25%) |
| G20 | Male | Deaths | 121776.28 | 83638.15 (68.68%) | 51139.33 (41.99%) | -13001.21 (-10.68%) |
| G20 | Female | Deaths | 60365.42 | 37127.35 (61.5%) | 23439.51 (38.83%) | -201.43 (-0.33%) |
| G20 | Both | Deaths | 182141.7 | 117817.83 (64.68%) | 74834.52 (41.09%) | -10510.64 (-5.77%) |
| China | Male | Deaths | 54158.94 | 59932.67 (110.66%) | 17359.67 (32.05%) | -23133.4 (-42.71%) |
| China | Female | Deaths | 22972.34 | 28214.67 (122.82%) | 7623.52 (33.19%) | -12865.85 (-56.01%) |
| China | Both | Deaths | 77131.28 | 88452.87 (114.68%) | 25423.38 (32.96%) | -36744.98 (-47.64%) |
| G20 | Male | DALYs (Disability-Adjusted Life Years) | 2606920.99 | 1987769.47 (76.25%) | 1512334.1 (58.01%) | -893182.59 (-34.26%) |
| G20 | Female | DALYs (Disability-Adjusted Life Years) | 1034252.78 | 735884.28 (71.15%) | 578801.51 (55.96%) | -280433.01 (-27.11%) |
| G20 | Both | DALYs (Disability-Adjusted Life Years) | 3641173.77 | 2683310.41 (73.69%) | 2100623.38 (57.69%) | -1142760.02 (-31.38%) |
| China | Male | DALYs (Disability-Adjusted Life Years) | 1239940.47 | 1556023.03 (125.49%) | 564706.26 (45.54%) | -880788.82 (-71.03%) |
| China | Female | DALYs (Disability-Adjusted Life Years) | 355218.24 | 608358.22 (171.26%) | 204163.03 (57.48%) | -457303.01 (-128.74%) |
| China | Both | DALYs (Disability-Adjusted Life Years) | 1595158.71 | 2191163.65 (137.36%) | 785572.24 (49.25%) | -1381577.17 (-86.61%) |
